# Overexpression of Rice Histone H1 Gene Reduces Tolerance to Cold and Heat Stress

**DOI:** 10.3390/plants12132408

**Published:** 2023-06-22

**Authors:** Jiale Wan, Jia Zhang, Xiaofei Zan, Jiali Zhu, Hao Chen, Xiaohong Li, Zhanmei Zhou, Xiaoling Gao, Rongjun Chen, Zhengjian Huang, Zhengjun Xu, Lihua Li

**Affiliations:** 1 Rice Research Institute, Sichuan Agricultural University, Chengdu 611130, China; 2 State Key Laboratory of Crop Gene Exploration and Utilization in Southwest China, Sichuan Agricultural University, Chengdu 611130, China; 3 Crop Ecophysiology and Cultivation Key Laboratory of Sichuan Province, Chengdu 611130, China

**Keywords:** rice, histone H1, cold stress, heat stress, agronomic traits

## Abstract

Temperature stresses, including low- and high-temperature stresses, are the main abiotic stresses affecting rice yield. Due to global climate change, the impact of temperature pressure on rice yield is gradually increasing, which is also a major concern for researchers. In this study, an H1 histone in *Oryza sativa* (*OsHis1.1*, LOC_Os04g18090) was cloned, and its role in rice’s response to temperature stresses was functionally characterized. The GUS staining analysis of *OsHis1.1* promoter-GUS transgenic rice showed that *OsHis1.1* was widely expressed in various rice tissues. Transient expression demonstrated that *OsHis1.1* was localized in the nucleus. The overexpression of *OsHis1.1* reduces the tolerance to temperature stress in rice by inhibiting the expression of genes that are responsive to heat and cold stress. Under stress conditions, the POD activity and chlorophyll and proline contents of *OsHis1.1*-overexpression rice lines were significantly lower than those of the wild type, while the malondialdehyde content was higher than that of the wild type. Compared with Nip, *OsHis1.1*-overexpression rice suffered more serious oxidative stress and cell damage under temperature stress. Furthermore, *OsHis1.1*-overexpression rice showed changes in agronomic traits.

## 1. Introduction

Rice (*Oryza sativa* L.) is one of the main food crops in the world. Crop growth and yield are seriously affected by abiotic stress, such as heat, cold, drought, waterlogging, and salinity. In recent years, with the intensification of global climate change, the adverse effects of abiotic stress have further expanded, especially those caused by high- and low-temperature stress [1,2,3].

Both cold and heat stress restrict the growth and development of plants, thus reducing grain yield [4,5,6]. A low temperature inhibits the metabolic reaction of plants and causes osmotic and oxidative stress, which are harmful to plants [7,8,9]. There are complex and diverse ways for plants to cope with low temperatures [7,10]. Extensive studies have shown that three C-repeat-binding factors/dehydration-responsive element-binding protein 1 (CBF/DREB1) play key roles in the cold acclimation of plants [11,12]. CBF transcription factors directly activate a series of Cold-Regulated (COR) genes that can increase freezing tolerance [6,10,13,14,15,16]. These COR genes generally encode some cryoprotective proteins, reactive oxygen species (ROS) scavenging proteins, enzymes for osmolyte biosynthesis and photosynthetic membrane protective proteins, which enhance the freezing resistance of plants [17,18,19]. Overexpressing *CBFs* increases the expression of COR genes and enhances the freezing tolerance of plants [20]. Heat stress can affect plants in many aspects, such as physiological and biochemical metabolism, gene expression and the fluidity of the cell membrane, and it results in the degradation of proteins, the inactivation of enzymes and the accumulation of ROS [5,21,22]. Heat stress disrupts electron transport, which enhances the production of ROS in chloroplasts and mitochondria [23]. The excessive accumulation of ROS will cause cell membrane damage and cell death [23,24]. The ROS scavenging ability of plants reflects their resistance to heat stress [21,25]. Another important way in which plants cope with heat stress is through the production of heat shock proteins (HSPs), many of which function as molecular chaperones to prevent protein denaturation and maintain protein homeostasis [26].

Nucleosomes make up the basic components of chromatin in eukaryotes. Nucleosomes are composed of DNA and core histones linked by histone H1 [27,28,29,30,31]. Histone H1 enhances the stability of nucleosomes [29]. At present, some studies have shown that histone H1 is involved in plants’ response to stress [29,30,31,32,33]. For example, the overexpression of the H1 histone gene in *Camellia sinensis* confers abiotic stress tolerance in transgenic tobacco [29]. However, a study on whether histone H1 in rice responds to abiotic stress has not been reported. In the present study, we found that the overexpression of the histone H1.1 gene (*OsHis1.1*) in rice (*Oryza sativa*) caused rice to be more sensitive to high and low temperatures and had a negative impact on the agronomic traits of rice.

## 2. Results

### 2.1. OsHis1.1 Sequence Analysis

**Figure 1:**
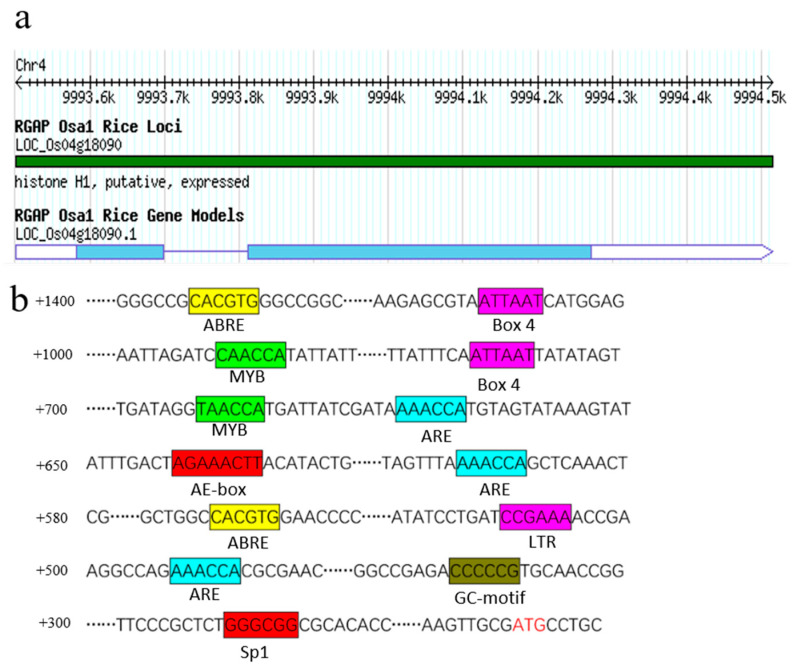
*OsHis1.1* sequence analysis. (**a**) *OsHis1.1* gene’s information. (**b**) Analysis of elements associated with the *OsHis1.1* promoter region.

*OsHis1.1* (LOC_Os04g18090) is located on rice chromosome 4 and has an open reading frame (ORF) sequence of 572bp, encoding 191 amino acids (Figure 1a). An approximately 1400 bp upstream sequence of the ATG was analyzed via PlantCARE, and it was found that it contained multiple elements associated with the stress response (Figure 1b). Besides the basic promoter element, these elements also include Box-4 (a cis-acting element involved in light response), ABRE (an abscisic acid cis-acting element), ARE (a cis-acting regulatory element essential for anaerobic induction) and the MYB response element.

### 2.2. Subcellular Localization of the OsHis1.1 Protein

**Figure 2:**
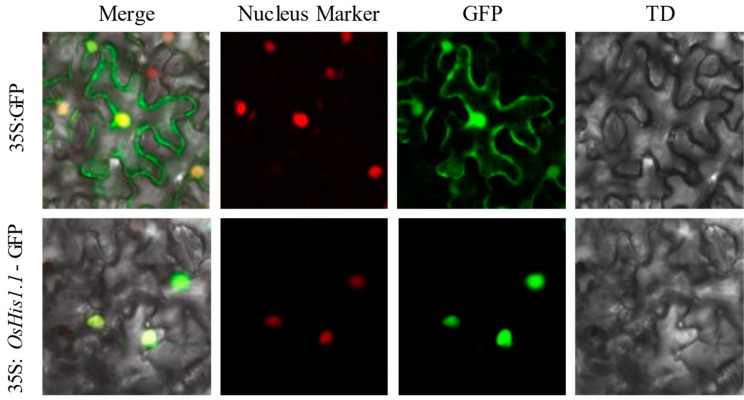
Subcellular localization of *OsHis1.1*–GFP in tobacco. 35S:GFP is the single GFP protein; 35S:*OsHis1.1*-GFP is the His1.1-GFP fusion protein.

The subcellular location of OsHis1.1 was experimentally verified via the transient transformation of CaMV35S-OsHis1.1-GFP in tobacco leaves. The green fluorescence signals of the green fluorescent protein (GFP) control were observed in the cell nucleus and the plasma membrane of the tobacco leaf, whereas the green fluorescence signals of the *OsHis1.1*:GFP fusion were only observed in the nucleus (Figure 2). The results showed that the *OsHis1.1* protein was located in the nucleus.

### 2.3. GUS Staining of Promoter Transgenic Plants

**Figure 3:**
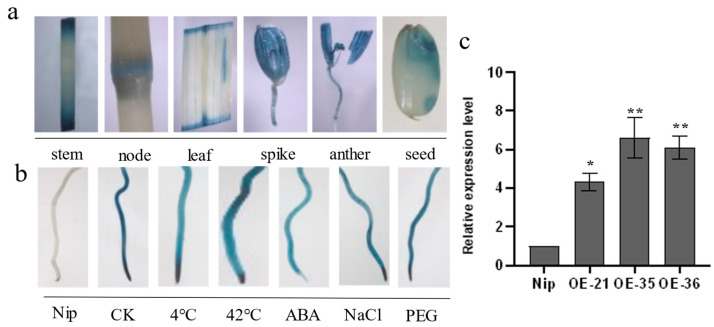
GUS staining of promoter transgenic plants and detection of *OsHis1.1* relative expression level. (**a**) *OsHis1.1* promoter-GUS transgenic plants showed staining of different tissues. (**b**) Detection of GUS activity in roots of *OsHis1.1* promoter-GUS transgenic plants under different stress treatments. (50 μM ABA, 180 mM NaCl and 20 % (*w*/*v*) PEG6000.) Nip is non-transformed wild-type rice. CK is *OsHis1.1* promoter-GUS transgenic rice. (**c)** Identification of *OsHis1.1*-overexpression rice lines. Error bars represent ±SE (*n* = 3). Asterisks indicate significant differences between transgenic lines and Nip (Dunnett’s test, * *p* < 0.05, ** *p* < 0.01).

To examine the expression pattern of *OsHis1.1*, the activity of β-glucuronidase (GUS) in *OsHis1.1* promoter-GUS transgenic rice plants was analyzed; the histochemical staining for GUS activity in various tissues was performed (Figure 3a); and strong GUS signals were observed in the stems (nodes and internodes), leaves, spikes, anthers and seeds. After exposing *OsHis1.1* promoter-GUS transgenic seedlings to different stress treatments, the GUS signal detected in the treatment group was significantly weaker than that in the control group (Figure 3b), further indicating that *OsHis1.1* may be involved in multiple stress responses.

### 2.4. Overexpression of OsHis1.1 Attenuated the Heat Stress Tolerance of Rice

**Figure 4:**
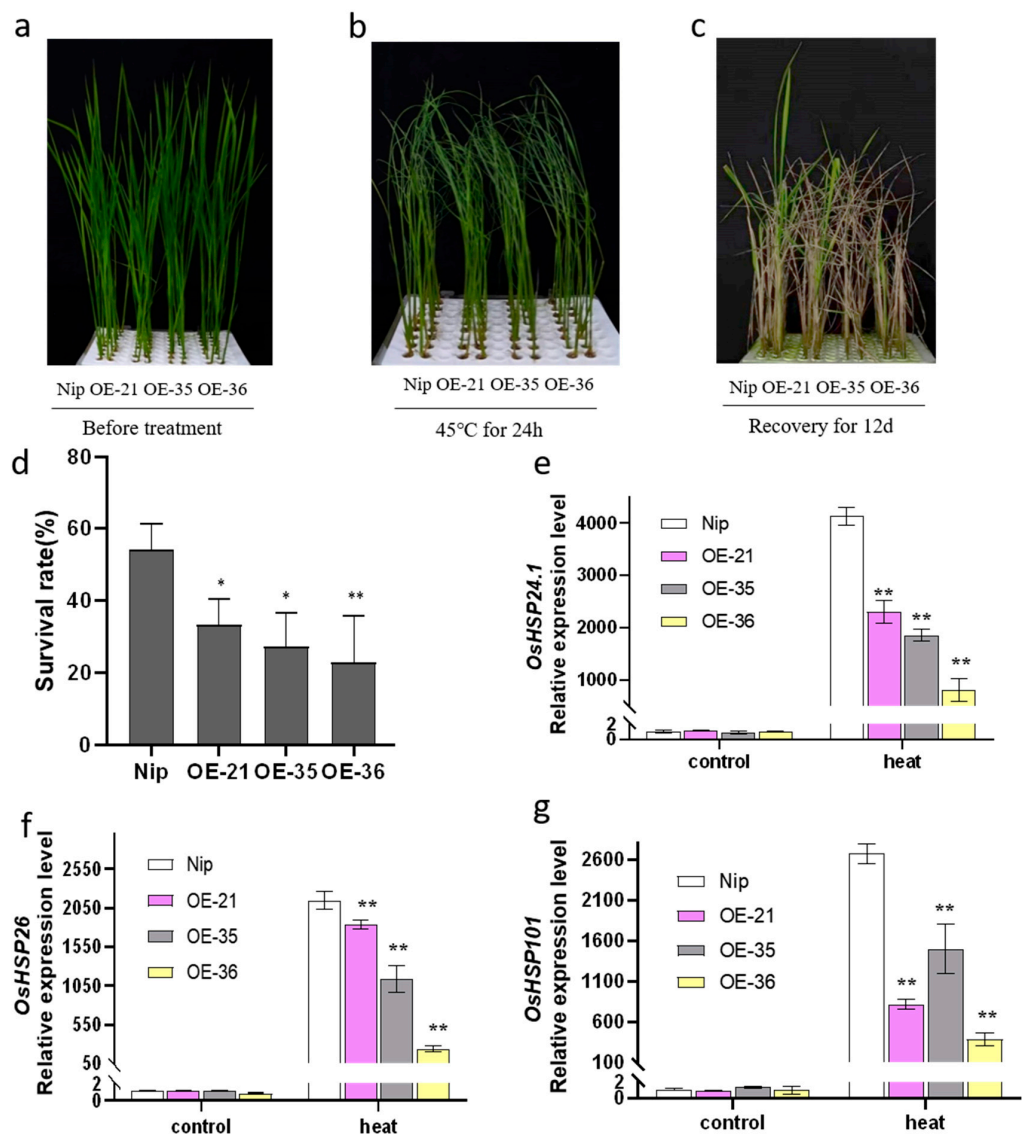
Detection of heat tolerance in *OsHis1.1*-overexpressed rice (**a**) Phenotype of WT and OE lines hydroponically cultivated for 14 days (before the imposition of the heat stress). (**b**) Phenotypes of WT and OE lines treated for 24 h at 45 °C. (**c**) Phenotypes of WT and OE lines after 12 d of recovery following the 45 °C treatment. (**d**) Survival rate of recovered WT and OE lines after heat stress. (Dunnett’s test, * *p* < 0.05, ** *p* < 0.01). (**e**–**g**) The expression levels of *OsHSP24.1*, *OsHSP26* and *OsHSP101* after 45 °C treatment. Error bars represent ±SE (*n* = 3). Asterisks indicate significant differences between transgenic lines and Nip (Dunnett’s test, * *p* < 0.05, ** *p* < 0.01).

In order to select overexpressed lines for subsequent experiments, a batch of rice lines was obtained through PCR detection and hygromycin selection (Figure S1). Then, the expression level of *OsHis1.1* was detected via RT-PCR (Figure 3c). We finally selected three overexpression rice lines (OE-21, OE-35 and OE-36). To verify the response of *OsHis1.1*-overexpression rice (*OsHis1.1*-OE) to heat stress, we cultured Nip and three overexpression lines (OE-21, OE-35 and OE-36) under normal conditions for 14 days (14 d) and then treated them at 45 °C for 1 d, and finally, we transferred the seedlings to normal conditions for 12 d (Figure 4a–c). The results showed that under normal conditions, the growth status of *OsHis1.1*-OE and Nip (wide-type rice) seedlings was almost the same, but after high-temperature treatment, the survival rate of *OsHis1.1*-OE seedlings was significantly lower than that of Nip (Figure 4d).

In order to explore the reasons for the decreased tolerance of *OsHis1.1*-OE to high-temperature stress, we detected the expression of important genes in the heat response pathway of rice. Under unstressed conditions, the expression levels of *OsHSP24.1*, *OsHSP26* and *OsHSP101* were no different between *OsHis1.1*-OE and Nip. Under heat stress, the expression levels of *OsHSP24.1*, *OsHSP26* and *OsHSP101* increased in *OsHis1.1*-OE and Nip, but the increase was significantly lower in *OsHis1.1*-OE than in Nip (Figure 4e–g).

### 2.5. Overexpression of OsHis1.1 Attenuated the Cold Stress Tolerance of Rice

**Figure 5:**
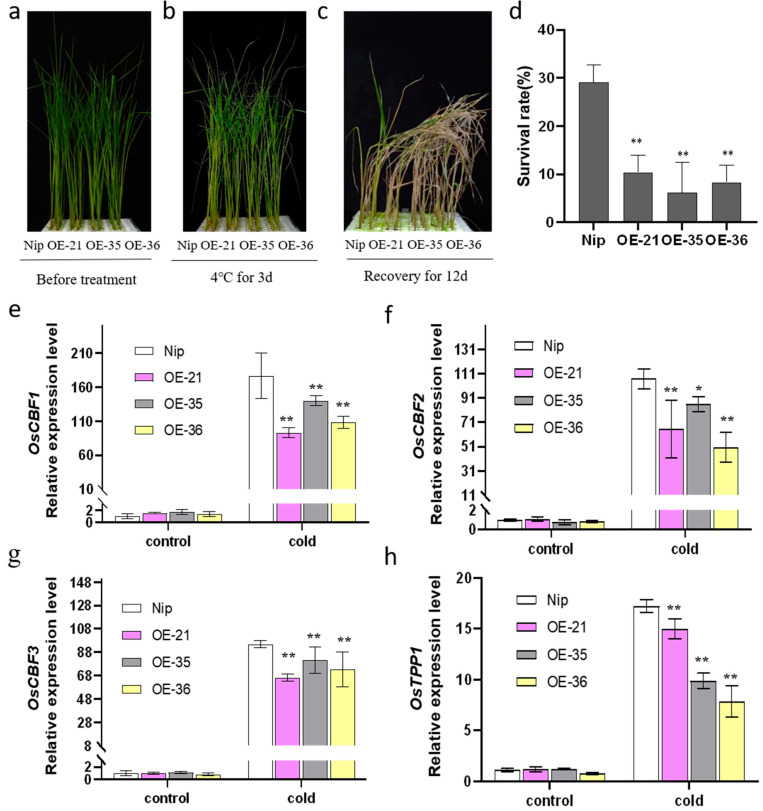
Detection of cold tolerance in *OsHis1.1*-overexpressed rice. (**a**) Phenotype of Nip and OE lines hydroponically cultivated for 14 days (before the imposition of the heat stress). (**b**) Phenotypes of Nip and OE lines treated for 3 d at 4 °C. (**c**) Phenotypes of Nip and OE lines after 12 d of recovery following the 4 °C treatment. (**d**) Survival rate of recovered Nip and OE lines after the 4 °C treatment. (Dunnett’s test, * *p* < 0.05, ** *p* < 0.01, *n* = 3). (**e**–**h**) The expression levels of *OsCBF1*, *OsCBF2, OsCBF3* and *OsTPP1* after 4 °C treatment. Error bars represent ±SE (*n* = 3). Asterisks indicate significant differences between transgenic lines and Nip (Dunnett’s test, * *p* < 0.05, ** *p* < 0.01).

To verify the response of *OsHis1.1*-OE to cold stress, we cultured Nip and three overexpression lines (OE-21, OE-35 and OE-36) under normal conditions for 14 d and then treated them at 4 °C for 3 d, and we finally transferred the seedlings to normal conditions for 12 d (Figure 5a–c). The results showed the growth statuses of *OsHis1.1*-OE and Nip seedlings under the normal condition were almost the same, but after low-temperature treatment, the survival rate of *OsHis1.1*-OE seedlings was significantly lower than that of Nip (Figure 5d).

In order to explore the reasons for the decreased tolerance of *OsHis1.1*-OE to low-temperature stress, we detected the expression of important genes in the cold response pathway of rice. The expression of *OsTPP1* has been shown to be positively correlated with the resistance to cold stress of rice. The overexpression of the trehalose-6-phosphate phosphatase gene *OsTPP1* in rice enhanced the tolerance to cold stress [34]. Under unstressed conditions, the expression levels of *OsCBF1*, *OsCBF2*, *OsCBF3* and *OsTPP1* were no different between *OsHis1.1*-OE and Nip. Under cold stress, the expression levels of *OsCBF1*, *OsCBF2*, *OsCBF3* and *OsTPP1* increased in *OsHis1.1*-OE and Nip, but the increase was significantly lower in *OsHis1.1*-OE than in Nip (Figure 5e–h).

### 2.6. Overexpression of OsHis1.1 Attenuated Oxidative Stress Resistance in Rice under Heat and Cold Stress

**Figure 6:**
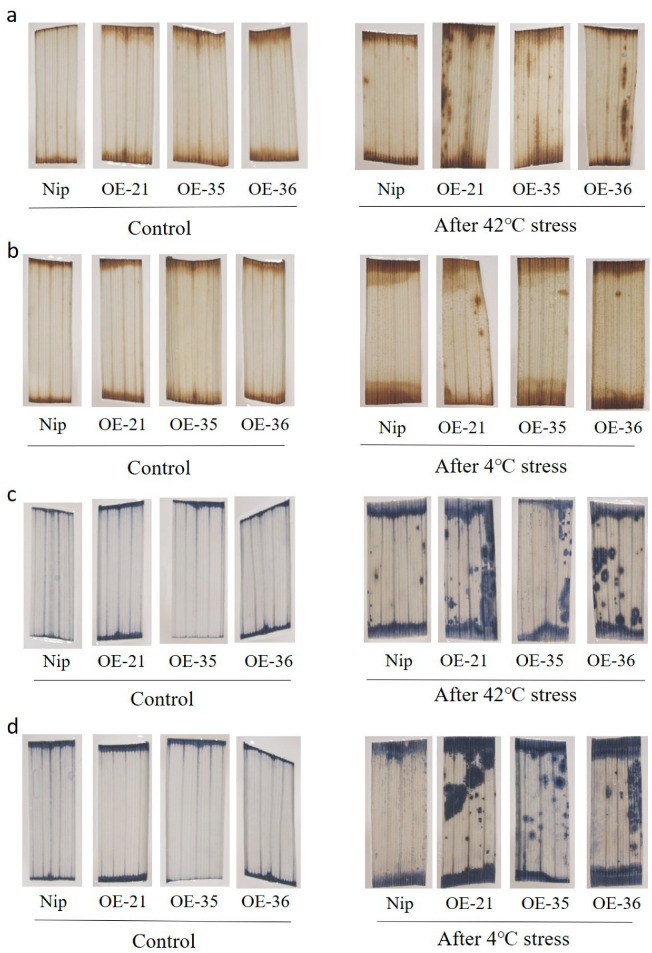
Accumulation of reactive oxygen species in *OsHis1.1* transgenic plants. (**a**,**b**) DAB staining was used to detect H_2_O_2_ in the Nip and OE lines before and after heat and cold treatments. (42 °C for 24 h and 4 °C for 2 d.) (**c**,**d**) NBT staining was used to detect the levels of superoxide anion in Nip and OE lines before and after heat and cold treatments. (42 °C for 24 h and 4 °C for 2 d.)

To investigate the effect of the overexpression of *OsHis1.1* on ROS accumulation, 14-day-old Nip, OE-21, OE-35 and OE-36 seedlings were subjected to heat and cold stress, and the accumulation of superoxide anion (O^2−^) and hydrogen peroxide (H_2_O_2_) was evaluated via Nitrotetrazolium Blue Chloride (NBT) and Diaminoaniline (DAB) staining, respectively. Under normal conditions, there were no obvious differences between the Nip and *OsHis1.1*-OE rice lines. After the cold and heat treatments, the results of NBT and DAB staining revealed that leaves of Nip accumulated less O^2−^ and H_2_O_2_ than *OsHis1.1*-OE lines (Figure 6a–d), which was reflected by less severe surface spots and browning surfaces in leaves of Nip (Figure 6a–d).

**Figure 7:**
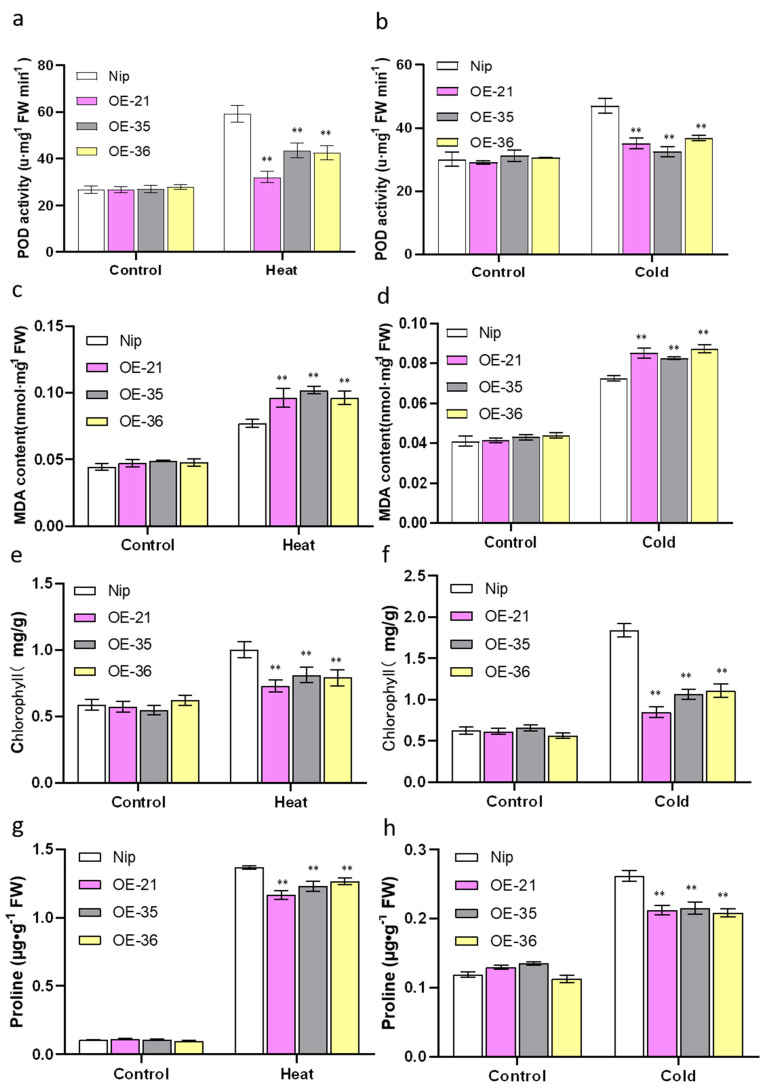
Determination of physiological parameters. (**a**,**b**) POD activities of Nip and OE lines before and after heat and cold stress (42 °C for 1 d and 4 °C for 2 d). (**c**,**d**) MDA contents of Nip and OE lines before and after heat and cold stress (42 °C for 1 d and 4 °C for 2 d). (**e**,**f**) Chlorophyll contents of Nip and OE lines before and after heat and cold stress (42 °C for 1 d and 4 °C for 2 d). (**g**,**h**) Proline contents of Nip and OE lines before and after heat and cold stress (42 °C for 1 d and 4 °C for 2 d). Error bars represent ±SE (*n* = 3). Asterisks indicate significant differences between OE lines and Nip (Dunnett’s test, ** *p* < 0.01).

In addition, the activities of peroxidase (POD) under heat and cold stress were detected. The results showed that there were no significant differences in the activities of POD between the *OsHis1.1*-OE lines and Nip under the normal conditions, whereas under the heat and cold stress conditions, the enzyme activity of Nip was higher than that of the *OsHis1.1*-OE lines (Figure 7a,b). We also found that the content of malondialdehyde (MDA) in Nip was significantly lower than that in the *OsHis1.1*-OE lines (Figure 7c,d), indicating that the *OsHis1.1*-OE lines suffered more serious cell membrane damages under stress condition [35,36]. These results further indicated that the *OsHis1.1*-OE rice accumulated more ROS than Nip under heat and cold stress.

### 2.7. Overexpression of OsHis1.1 Reduced Chlorophyll and Proline Contents in Rice under Heat and Cold Stress

Chlorophyll content can be used as an important indicator to judge the tolerance of plants to stress [37,38]. And proline (Pro) is not only an osmotic regulation substance, but also a metal chelating agent and antioxidant defense molecule [39]. The accumulation of Pro is a protective response of plants to environmental stress [40]. Under normal growth conditions, the Pro and chlorophyll contents of the *OsHis1.1*-OE lines did not differ from those of Nip. Under stress conditions, both the *OsHis1.1-OE* lines and the wild type (Nip) showed increased Pro and chlorophyll content. However, under the same stress treatment, Pro and chlorophyll contents were reduced in the *OsHis1.1*-OE lines compared to Nip (Figure 7e–h). The above results indicate that the overexpression of *OsHis1.1* could decrease the content of Pro and chlorophyll, thus reducing heat and cold stress tolerance.

### 2.8. Overexpression of OsHis1.1 Affects the Agronomic Traits of Rice

**Figure 8:**
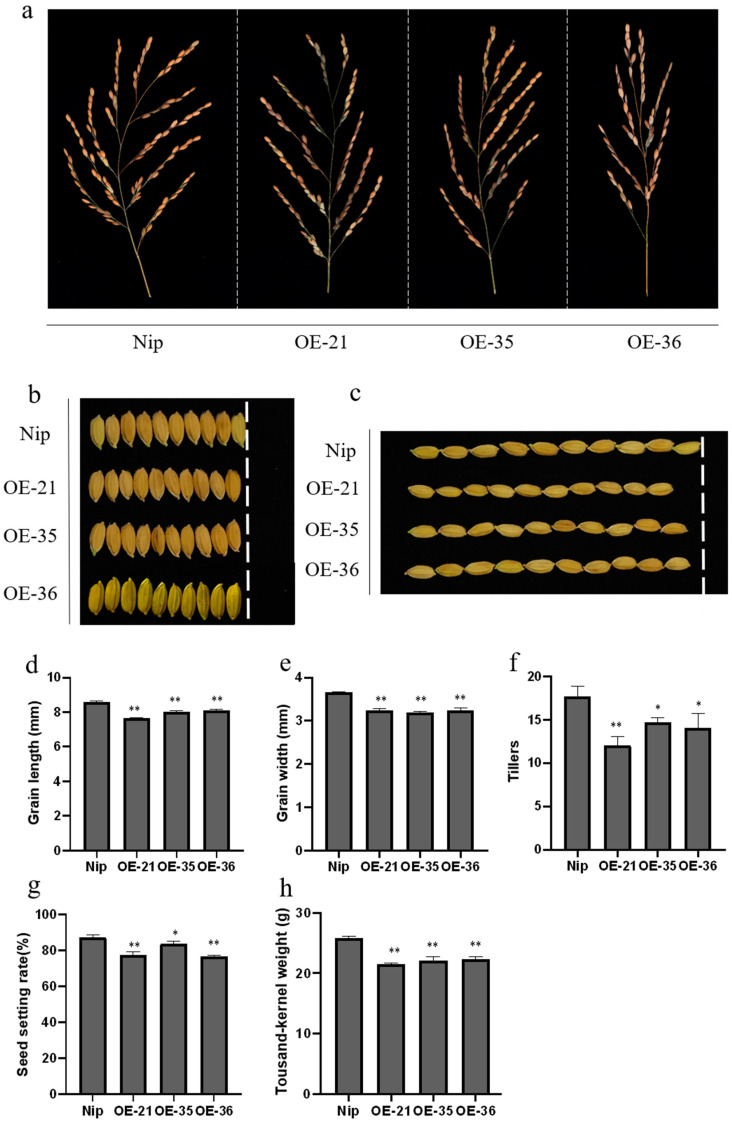
Determination of agronomic traits. (**a**–**c**) Phenotypes of Nip and OE lines in rice panicle, grain length and grain width. (**d**–**h**) Statistics of agronomic traits of Nip and OE lines in grain length, grain width, tiller, seed setting rate and thousand-kernel weight. Error bars represent ±SE (*n* = 3). Asterisks indicate significant differences between OE lines and Nip (Dunnett’s test, * *p* < 0.05, ** *p* < 0.01).

In this study, related agronomic traits were observed in *OsHis1.1*-OE and Nip (Figure 8a–c). Compared with Nip, the length and width of grain, 1000-grain weight, seed setting rate and the number of tillers were all lower in *OsHis1.1*-OE lines (Figure 8d–h).

## 3. Discussion

As a linker protein, histone H1 works as the structural component of chromatin in both plants and animals [41,42]. It localizes to the nucleus in plant cells, and in this study, we also found that the *OsHis1.1* protein localizes to the nucleus, which is consistent with previous results [29,33,43]. In recent years, as research has continued, there has been increasing experimental evidence that histone H1 plays an important role in abiotic stress responses, in addition to being a structural component [29,30,33]. The expression of histone H1 in *Camellia sinensis* is induced by low temperatures [29]. Histone H1 could be induced by ABA and drought in tomato and Arabidopsis [30,44]. However, there are few studies on H1’s response to temperature stress. This study showed that histone H1 in rice affected the tolerance of rice to low and high temperatures.

The ROS is one of the important products in plants’ abiotic stress response, and a change in its content is closely related to stress tolerance. Under normal conditions, the production and scavenging of ROS in plants are balanced, but when faced with abiotic stresses, this balance is disrupted, and the excessive accumulation of ROS is toxic to the plant [24,45,46]. POD is an important ROS-scavenging enzyme in plants, which can help plants resist peroxidation [24]. *OsANN10* RNAi lines reduced the damage made by ROS through increasing the activity of POD [47]. The overexpression of *OsMADS23* could increase the activities of POD and CAT under osmotic stress [48]. In our results, *OsHis1.1*-OE lines accumulated more ROS than Nip under heat and cold stress, which could be confirmed by the DAB and NBT staining results. Meanwhile, the activity of POD was lower in *OsHis1.1*-OE lines than in Nip.

MDA is an indicator of the degree of cell membrane damage by ROS. In this study, after heat and cold stress, the content of MDA in *OsHis1.1*-OE was higher than in Nip. Pro, an important amino acid, plays an important role in maintaining the metabolism and growth of plants under abiotic stress conditions [49] and has been proposed to enhance tolerance to abiotic stress in rice [50]. In our study, the content of Pro was lower in *OsHis1.1*-OE than in Nip after heat and cold stress, which was consistent with the stress phenotype. Thus, we speculated that the overexpression of *OsHis1.1* could aggravate damage caused by heat and cold stress by affecting the activity of related enzymes and the accumulation of osmotic substances and MDA, further decreasing heat and cold stress tolerance.

Regulating stress tolerance by regulating the expression of stress-related genes is one of the important ways for plants to cope with abiotic stresses. HSPs are important for plants to cope with heat stress [51]. It has been proved that the expression levels of *HSP24.1*, *HSP26* and *HSP101* are positively correlated with the heat tolerance of plants [51,52,53]. The overexpression of the *AtHSP101* gene of Arabidopsis in rice enhanced the heat tolerance of rice [53]. CBF transcription factors are involved in the cold signaling pathway in plants, and as one of the soluble sugar components, trehalose plays an important role in the cold stress response. The overexpression of *AtCBF1* in potato induces cold-acclimation-associated physiological modifications [54]. In our results, under heat stress conditions, the expression levels of *OsHSP24.1*, *OsHSP101* and *OsHSP26* were all significantly lower in *OsHis1.1*-OE lines than those in Nip. Meanwhile, under cold stress conditions, similar results were observed in the expression levels of *OsCBF1*, *OsCBF2*, *OsCBF3* and *OsTPP1* between *OsHis1.1*-OE and Nip. These results imply, therefore, that the overexpression of *OsHis1.1* could decrease the heat and cold tolerance of rice by affecting the transcript accumulation of these stress-responsive genes. The probable reason for this is that the overexpression of histone H1 leads to slow DNA unpacking under stress conditions.

Grain size (grain length and width) and grain weight are important agronomic traits in crop production. In our results, we compared some agronomic traits of *OsHis1.1*-OE and Nip and found that Nip had better agronomic traits than *OsHis1.1*-OE. The decrease in these traits of *OsHis1.1*-OE rice would result in lower yields than Nip. These results are consistent with previous studies that showed histone H1 represses gene expression and histone H1 is crucial to many life processes including development, differentiation and apoptosis [55,56,57].

## 4. Materials and Methods

### 4.1. Plant Materials, Growth Conditions and Abiotic Stress Treatments

The plant material *Oryza sativa* L. subsp. *japonica* cv. Nipponbare (Nip) was used in this experiment as the wild-type rice. *OsHis1.1* promoter-GUS transgenic rice plants and *OsHis1.1*-OE transgenic rice plants were generated in the Nip background. Screened with hygromycin and PCR detection (Figure S1), T_2_ generation plants were used in this experiment [58]. All methods were performed in accordance with the relevant guidelines and regulations.

Rice plants grew in nutrient solution and were placed in an artificial climate incubator at 28 °C/22 °C and with a 16 h light/8 h dark cycle. After 2 weeks of cultivation, 20 consistent seedlings for each rice line were selected and stress treatments were conducted: heat treatment (45 °C) for 1 day and cold treatment (4 °C) for 3 days. After undergoing the stress treatment and being transferred to an unstressed environment, seedlings whose leaves remained wilted after 12 days were considered dead, while seedlings that turned green were those that survived.

### 4.2. Analysis and Cloning of the OsHis1.1 Gene

The *OsHis1.1* gene information was obtained from the Rice Genome Annotation Project (RGAP, http://rice.uga.edu/, accessed on 10 May 2020) and National Center for Biotechnology Information (NCBI, http://www.ncbi.nlm.nih.gov/, accessed on 10 May 2020) database. A polymerase chain reaction (PCR) was used to amplify the *OsHis1.1* and promoter sequence (about 1500 bp upstream of the ATG). All primers are listed in Table S1.

### 4.3. Plasmid Construction and Rice Transformation

To generate the *OsHis1.1*-overexpression lines, the entire *OsHis1.1* coding region was connected to the vector pCAMBIA1300 (a vector with some modifications; primer: OsH1.1-OE-F and OsH1.1-OE-R). To generate *OsHis1.1* promoter-GUS transgenic rice plants, the promoter sequence was connected to the vector pCAMBIA1305 (primer: OsH1.1-GUS-F and OsH1.1-GUS-R). The *OsHis1.1* gene was linked to the pCAMBIA1300-GFP (with some modifications; primer: OsH1.1-GFP-F and OsH1.1-GFP-R) vector for subcellular localization. These constructs were introduced into Nip via the Agrobacterium-mediated transformation method [59].

### 4.4. Subcellular Localization and GUS Activity Analysis

The fusion construct CaMV 35S: *OsHis1.1*-GFP and empty vector CaMV 35S:GFP were transiently transformed into tobacco (*Nicotiana benthamiana*) leaves, and the fluorescence was observed via confocal microscopy after 48 h.

The method described by Jefferson was used to detect GUS activity using histochemical staining [60]. Different tissues of *OsHis1.1* promoter-GUS transgenic rice were placed in a buffer containing 50 mM sodium phosphate at pH 7.0, 10 mM EDTA, 5 mM K_3_Fe (CN)_6_, 5 mM K_4_Fe (CN)_6_, 0.1% (*w*/*w*) Triton-100 and 1 mg/mL X-Gluc, and they were incubated overnight at 37 °C [31]. The tissues were then soaked in 70% (*v*/*v*) ethanol for 5 min to stop the staining; then, 95% (*v*/*v*) ethanol was added and boiled until the chlorophyll was completely removed. Finally, photos were taken with a ZEISS stereo microscope (Carl Zeiss AG, Oberkochen, Germany).

### 4.5. Isolation of DNA and RNA and Real-Time PCR

Fresh leaves were sampled at different time periods and immediately frozen in liquid nitrogen. Genomic DNA was extracted from rice seedlings via the CTAB method [61].

The total RNA was extracted using Trizol reagent (Invitrogen, Burlington, ON, Canada) according to the manufacturer’s protocol. The reverse transcription was conducted using a PrimeScript™ RT Reagent kit with a gDNA Eraser kit (+GDNA wiperVazyme, Beijing, China), and the cDNA was stored at −20 °C. The relative expression levels of target genes were determined based on the 2^−△△C^ method, and the Ubiquitin gene of rice (LOC_Os01g22490) was used as an internal control [62]. All primers are listed in Supplementary Table S1.

### 4.6. Measurement of the Physiological Parameters

The two-week-old seedlings were used for two different treatments. After the completion of the treatments, the physiological and biochemical indicator parameters of plants were determined. The method described by Surender Reddy was used to detect the content of MDA and Pro [63]. The method described by Gao was used to detect the chlorophyll content [47], and the method described by Chen was used to detect the activity of antioxidant enzyme POD [64]. Leaves were placed in 1 mg/mL DAB and 6 mM NBT staining solution and incubated at 28 °C for 10 h in light [65]. Anhydrous ethanol was used to remove chlorophyll. The accumulation of hydrogen peroxide and superoxide anion O_2_^−^ was observed under a stereo microscope (Carl Zeiss AG, Oberkochen, Germany).

## 5. Conclusions

In this study, we found that the histone H1 gene, *OsHis1.1*, affects the response of rice to high and low temperatures. The overexpression of *OsHis1.1* suppressed the expression of genes responsive to temperature stress, resulting in reduced tolerance to cold and heat stress in rice. Compared to Nip, *OsHis1.1*-OE rice suffered more severe oxidative stress and cellular damage under temperature stress. The overexpression of *OsHis1.1* altered the agronomic traits of rice.

## Data Availability

The *OsHis1.1* gene information is available in the National Center for Biotechnology Information (NCBI) repository (XM_015781730.2). The *OsHis1.1* gene promoter sequence is available in the NCBI repository (NC_029259.1). The datasets supporting the findings of this article are included within the article and its additional files. The data are available from the corresponding author on reasonable request.

## Supplementary Materials

The following supporting information can be downloaded at: https://www.mdpi.com/article/10.3390/plants12132408/s1.

## References

[1] Jiang J., Bai J., Li S., Li X., Yang L., He Y. (2018). *HTT2* Promotes Plant Thermotolerance in Brassica Rapa. BMC Plant Biol..

[2] Shekhawat K., Saad M.M., Sheikh A., Mariappan K., Al-Mahmoudi H., Abdulhakim F., Eida A.A., Jalal R., Masmoudi K., Hirt H. (2021). Root Endophyte Induced Plant Thermotolerance by Constitutive Chromatin Modification at Heat Stress Memory Gene Loci. EMBO Rep..

[3] Lyman N.B., Jagadish K.S., Nalley L.L., Dixon B.L., Siebenmorgen T. (2013). Neglecting Rice Milling Yield and Quality Underestimates Economic Losses from High-Temperature Stress. PLoS ONE.

[4] Zhang P., Qian D., Luo C., Niu Y., Li T., Li C., Xiang Y., Wang X., Niu Y. (2021). Arabidopsis *ADF5* Acts as a Downstream Target Gene of Cbfs in Response to Low-Temperature Stress. Front. Cell Dev. Biol..

[5] Huang L.Z., Zhou M., Ding Y.F., Zhu C. (2022). Gene Networks Involved in Plant Heat Stress Response and Tolerance. Int. J. Mol. Sci..

[6] Thomashow M.F. (1999). Plant Cold Acclimation: Freezing Tolerance Genes and Regulatory Mechanisms. Annu. Rev. Plant Physiol. Plant Mol. Biol..

[7] Diao P., Chen C., Zhang Y., Meng Q., Lv W., Ma N. (2020). The Role of *NAC* Transcription Factor in Plant Cold Response. Plant Signal. Behav..

[8] Yuan P., Yang T., Poovaiah B.W. (2018). Calcium Signaling-Mediated Plant Response to Cold Stress. Int. J. Mol. Sci..

[9] Ye K., Li H., Ding Y., Shi Y., Song C., Gong Z., Yang S. (2019). *BRASSINOSTEROID-INSENSITIV2* Negatively Regulates the Stability of Transcription Factor *ICE1* in Response to Cold Stress in Arabidopsis. Plant Cell.

[10] Li H., Ye K., Shi Y., Cheng J., Zhang X., Yang S. (2017). *BZR1* Positively Regulates Freezing Tolerance Via *CBF*-Dependent and *CBF*-Independent Pathways in Arabidopsis. Mol. Plant.

[11] Zhou Y., Chen M., Guo J., Wang Y., Min D., Jiang Q., Ji H., Huang C., Wei W., Xu H. (2020). Overexpression of Soybean *DREB1* Enhances Drought Stress Tolerance of Transgenic Wheat in the Field. J. Exp. Bot..

[12] Moon S.J., Min M.K., Kim J.A., Kim D.Y., Yoon I.S., Kwon T.R., Byun M.O., Kim B.G. (2019). Ectopic Expression of Osdreb1g, a Member of the *OsDREB1* Subfamily, Confers Cold Stress Tolerance in Rice. Front. Plant Sci..

[13] Stockinger E.J., Gilmour S.J., Thomashow M.F. (1997). *Arabidopsis thaliana CBF1* Encodes an AP2 Domain-Containing Transcriptional Activator That Binds to the C-Repeat/DRE, a Cis-Acting DNA Regulatory Element That Stimulates Transcription in Response to Low Temperature and Water Deficit. Proc. Natl. Acad. Sci. USA.

[14] Shi Y., Ding Y., Yang S. (2015). Cold Signal Transduction and Its Interplay with Phytohormones During Cold Acclimation. Plant Cell Physiol..

[15] Agarwal M., Hao Y., Kapoor A., Dong C.-H., Fujii H., Zheng X., Zhu J.-K. (2006). A R2R3 Type Myb Transcription Factor Is Involved in the Cold Regulation of CBF Genes and in Acquired Freezing Tolerance. J. Biol. Chem..

[16] Liu Q., Kasuga M., Sakuma Y., Abe H., Miura S., Yamaguchi-Shinozaki K., Shinozaki K. (1998). Two Transcription Factors, DREB1 and DREB2, with an EREBP/AP2 DNA Binding Domain Separate Two Cellular Signal Transduction Pathways in Drought- and Low-Temperature-Responsive Gene Expression, Respectively, in Arabidopsis. Plant Cell.

[17] Li H., Ding Y., Shi Y., Zhang X., Zhang S., Gong Z., Yang S. (2017). MPK3- and MPK6-Mediated ICE1 Phosphorylation Negatively Regulates ICE1 Stability and Freezing Tolerance in Arabidopsis. Dev. Cell.

[18] Jia Y., Ding Y., Shi Y., Zhang X., Gong Z., Yang S. (2016). The CBFs Triple Mutants Reveal the Essential Functions of CBFs in Cold Acclimation and Allow the Definition of CBF Regulons in Arabidopsis. New Phytol..

[19] Chinnusamy V., Ohta M., Kanrar S., Lee B.H., Hong X., Agarwal M., Zhu J.K. (2003). ICE1: A Regulator of Cold-Induced Transcriptome and Freezing Tolerance in Arabidopsis. Genes Dev..

[20] Tang K., Zhao L., Ren Y., Yang S., Zhu J.K., Zhao C. (2020). The Transcription Factor ICE1 Functions in Cold Stress Response by Binding to the Promoters of Cbf and Cor Genes. J. Integr. Plant Biol..

[21] Sharma L., Priya M., Kaushal N., Bhandhari K., Chaudhary S., Dhankher O.P., Prasad P.V.V., Siddique K.H.M., Nayyar H. (2020). Plant Growth-Regulating Molecules as Thermoprotectants: Functional Relevance and Prospects for Improving Heat Tolerance in Food Crops. J. Exp. Bot..

[22] Wang X., Hou L., Lu Y., Wu B., Gong X., Liu M., Wang J., Sun Q. (2018). Vierling, and S. Xu. Metabolic Adaptation of Wheat Grain Contributes to a Stable Filling Rate under Heat Stress. J. Exp. Bot..

[23] Asthir B. (2015). Protective Mechanisms of Heat Tolerance in Crop Plants. J. Plant Interact..

[24] Nadarajah K.K. (2020). Ros Homeostasis in Abiotic Stress Tolerance in Plants. Int. J. Mol. Sci..

[25] Rashmi A., Bhandari K., Nayyar H. (2015). Temperature Stress and Redox Homeostasis in Agricultural Crops. Front. Environ. Sci..

[26] Zhu J.K. (2016). Abiotic Stress Signaling and Responses in Plants. Cell.

[27] Harshman S.W., Young N.L., Parthun M.R., Freitas M.A. (2013). H1 Histones: Current Perspectives and Challenges. Nucleic Acids Res..

[28] Andres M., Garcia-Gomis D., Ponte I., Suau P., Roque A. (2020). Histone H1 Post-Translational Modifications: Update and Future Perspectives. Int. J. Mol. Sci..

[29] Wang W., Wang Y., Du Y., Zhao Z., Zhu X., Jiang X., Shu Z., Yin Y., Li X. (2014). Overexpression of *Camellia Sinensis* H1 Histone Gene Confers Abiotic Stress Tolerance in Transgenic Tobacco. Plant Cell Rep..

[30] Scippa G.S., Griffiths A., Chiatante D., Bray E.A. (2000). The H1 Histone Variant of Tomato, H1-S, Is Targeted to the Nucleus and Accumulates in Chromatin in Response to Water-Deficit Stress. Planta.

[31] Rutowicz K., Puzio M., Halibart-Puzio J., Lirski M., Kotlinski M., Kroten M.A., Knizewski L., Lange B., Muszewska A., Sniegowska-Swierk K. (2015). A Specialized Histone H1 Variant Is Required for Adaptive Responses to Complex Abiotic Stress and Related DNA Methylation in Arabidopsis. Plant Physiol..

[32] Trivedi I., Ranjan A., Sharma Y.K., Sawant S. (2012). The Histone H1 Variant Accumulates in Response to Water Stress in the Drought Tolerant Genotype of *Gossypium herbaceum* L. Protein J..

[33] Wang J.N., Kuang J.F., Shan W., Chen J., Xie H., Lu W.J., Chen J.W., Chen J.Y. (2012). Expression Profiles of a Banana Fruit Linker Histone H1 Gene *MaHis1* and Its Interaction with a WRKY Transcription Factor. Plant Cell Rep..

[34] Ge L.F., Chao D.Y., Shi M., Zhu M.Z., Gao J.P., Lin H.X. (2008). Overexpression of the Trehalose-6-Phosphate Phosphatase Gene *OsTPP1* Confers Stress Tolerance in Rice and Results in the Activation of Stress Responsive Genes. Planta.

[35] Goswami A., Banerjee R., Raha S. (2013). Drought Resistance in Rice Seedlings Conferred by Seed Priming: Role of the Anti-Oxidant Defense Mechanisms. Protoplasma.

[36] Liu W., Yu K., He T., Li F., Zhang D., Liu J. (2013). The Low Temperature Induced Physiological Responses of *Avena nuda* L., a Cold-Tolerant Plant Species. Sci. World J..

[37] Jespersen D., Zhang J., Huang B. (2016). Chlorophyll Loss Associated with Heat-Induced Senescence in Bentgrass. Plant Sci..

[38] Song Y., Feng L., Alyafei M.A.M., Jaleel A., Ren M. (2021). Function of Chloroplasts in Plant Stress Responses. Int. J. Mol. Sci..

[39] Zuo Z.-F., Kang H.-G., Park M.-Y., Jeong H., Sun H.-J., Yang D.-H., Lee Y.-E., Song P.-S., Lee H.-Y. (2019). Overexpression of *ICE1*, a Regulator of Cold-Induced Transcriptome, Confers Cold Tolerance to Transgenic Zoysia Japonica. J. Plant Biol..

[40] Zarea M.J., Karimi N. (2023). Zinc-Regulated P5cs and Sucrose Transporters *SUT1B* Expression to Enhance Drought Stress Tolerance in Wheat. J. Plant Growth Regul..

[41] Ascenzi R., Gantt J.S. (1999). Subnuclear Distribution of the Entire Complement of Linker Histone Variants in *Arabidopsis thaliana*. Chromosoma.

[42] Breneman J.W., Yau P., Teplitz R.L., Bradbury E.M. (1993). A Light Microscope Study of Linker Histone Distribution in Rat Metaphase Chromosomes and Interphase Nuclei. Exp. Cell Res..

[43] Tanaka I., Akahori Y., Gomi K., Suzuki T., Ueda K. (1999). A Novel Histone Variant Localized in Nucleoli of Higher Plant Cells. Chromosoma.

[44] Ascenzi R., Gantt J.S. (1997). A Drought-Stress-Inducible Histone Gene in *Arabidopsis thaliana* Is a Member of a Distinct Class of Plant Linker Histone Variants. Plant Mol. Biol..

[45] Gill S.S., Tuteja N. (2010). Reactive Oxygen Species and Antioxidant Machinery in Abiotic Stress Tolerance in Crop Plants. Plant Physiol. Biochem..

[46] Das K., Roychoudhury A. (2014). Reactive Oxygen Species (Ros) and Response of Antioxidants as Ros-Scavengers During Environmental Stress in Plants. Front. Environ. Sci..

[47] Gao S., Song T., Han J., He M., Zhang Q., Zhu Y., Zhu Z. (2020). A Calcium-Dependent Lipid Binding Protein, *OsANN10*, Is a Negative Regulator of Osmotic Stress Tolerance in Rice. Plant Sci..

[48] Li X., Yu B., Wu Q., Min Q., Zeng R., Xie Z., Huang J. (2021). OsRKY23 Phosphorylated by SAPK9 Confers Drought and Salt Tolerance by Regulating Aba Biosynthesis in Rice. PLoS Genet..

[49] Ghosh U.K., Islam M.N., Siddiqui M.N., Cao X., Khan M.A.R. (2022). Proline, a Multifaceted Signalling Molecule in Plant Responses to Abiotic Stress: Understanding the Physiological Mechanisms. Plant Biol..

[50] Guo Z., Cai L., Liu C., Chen Z., Guan S., Ma W., Pan G. (2022). Low-Temperature Stress Affects Reactive Oxygen Species, Osmotic Adjustment Substances, and Antioxidants in Rice (*Oryza sativa* L.) at the Reproductive Stage. Sci. Rep..

[51] Xue Y., Peng R., Xiong A., Li X., Zha D., Yao Q. (2010). Over-Expression of Heat Shock Protein Gene *Hsp26* in *Arabidopsis thaliana* Enhances Heat Tolerance. Biol. Plant..

[52] Rezaul I.M., Baohua F., Tingting C., Weimeng F., Caixia Z., Longxing T., Guanfu F. (2019). Abscisic Acid Prevents Pollen Abortion under High-Temperature Stress by Mediating Sugar Metabolism in Rice Spikelets. Physiol. Plant.

[53] Katiyar-Agarwal S., Agarwal M., Grover A. (2003). Heat-Tolerant Basmati Rice Engineered by over-Expression of *Hsp101*. Plant Mol. Biol..

[54] Pino M.T., Skinner J.S., Jeknić Z., Hayes P.M., Soeldner A.H., Thomashow M.F., Chen T.H. (2008). Ectopic *AtCBF1* over-Expression Enhances Freezing Tolerance and Induces Cold Acclimation-Associated Physiological Modifications in Potato. Plant Cell Environ..

[55] Laybourn P.J., Kadonaga J.T. (1991). Role of Nucleosomal Cores and Histone H1 in Regulation of Transcription by RNA Polymerase Ii. Science.

[56] Flanagan T.W., Brown D.T. (2016). Molecular Dynamics of Histone H1. Biochim. Biophys. Acta (BBA)—Gene Regul. Mech..

[57] Yellajoshyula D., Brown D.T. (2006). Global Modulation of Chromatin Dynamics Mediated by Dephosphorylation of Linker Histone H1 Is Necessary for Erythroid Differentiation. Proc. Natl. Acad. Sci. USA.

[58] Zhang A., Liu Y., Wang F., Li T., Chen Z., Kong D., Bi J., Zhang F., Luo X., Wang J. (2019). Enhanced Rice Salinity Tolerance Via CRISPR/Cas9-Targeted Mutagenesis of the *OsRR22* Gene. Mol. Breed..

[59] Hiei Y., Ohta S., Komari T., Kumashiro T. (1994). Efficient Transformation of Rice (*Oryza sativa* L.) Mediated by Agrobacterium and Sequence Analysis of the Boundaries of the T-DNA. Plant J..

[60] Jefferson R.A. (1989). The Gus Reporter Gene System. Nature.

[61] Stewart C.N., Via L.E. (1993). A Rapid Ctab DNA Isolation Technique Useful for Rapd Fingerprinting and Other PCR Applications. Biotechniques.

[62] Livak K.J., Schmittgen T.D. (2001). Analysis of Relative Gene Expression Data Using Real-Time Quantitative Pcr and the 2−Δδct Method. Methods.

[63] Surender Reddy P., Jogeswar G., Rasineni G.K., Maheswari M., Reddy A.R., Varshney R.K., Kishor P.B.K. (2015). Proline over-Accumulation Alleviates Salt Stress and Protects Photosynthetic and Antioxidant Enzyme Activities in Transgenic Sorghum [*Sorghum bicolor* (L.) Moench]. Plant Physiol. Biochem..

[64] Chen T., Li W., Hu X., Guo J., Liu A., Zhang B. (2015). A Cotton Myb Transcription Factor, *GbMYB5*, Is Positively Involved in Plant Adaptive Response to Drought Stress. Plant Cell Physiol..

[65] Jambunathan N. (2010). Determination and Detection of Reactive Oxygen Species (Ros), Lipid Peroxidation, and Electrolyte Leakage in Plants. Methods Mol. Biol..

