# Supplementary material for: Overexpression of Rice Histone H1 Gene Reduces Tolerance to Cold and Heat Stress
Source: Plants (Basel). 2023 Jun 22;12(13):2408. doi: 10.3390/plants12132408 (PMC10346724; doi:10.3390/plants12132408)
Supplement: Supplementary file 1 [file plants-12-02408-s001.zip › plants-2462830-supplementary.pdf]

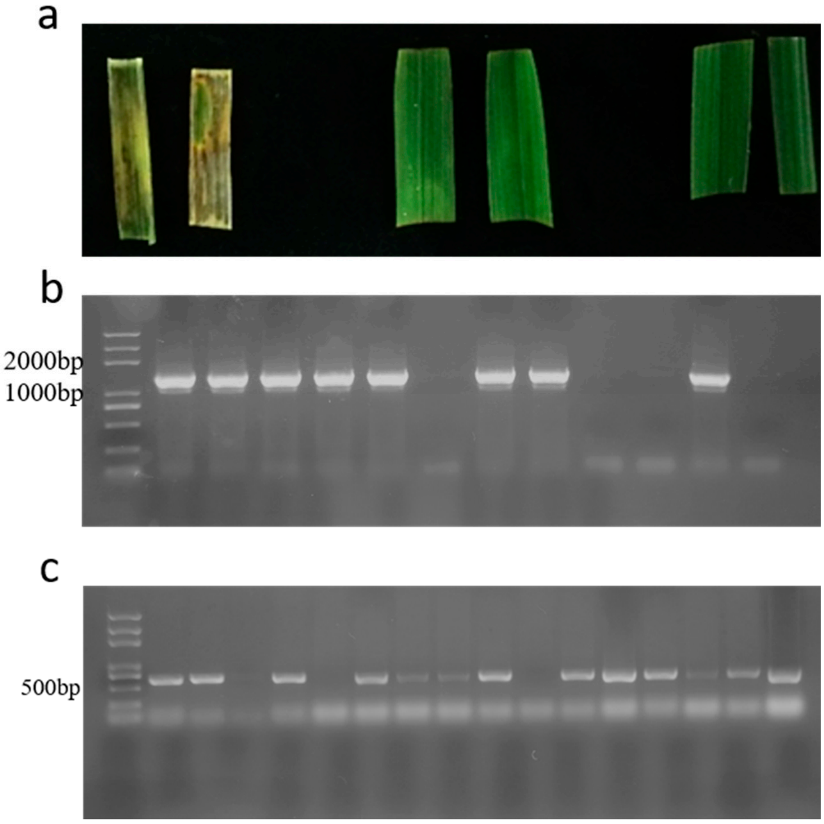

Figure S1. Detection of transgenic rice (a) Transgenic rice was screened with hygromycin. (Wild-type on the left, *OsHis1.1* promoter-GUS transgenic rice in the middle and OsHis1.1-OE transgenic rice on the right). (b) Detection of *OsHis1.1* promoter-GUS transgenic rice (primer: OsH1.1-GUS-F and GUS-R). (c) Detection of OsHis1.1-OE transgenic rice (primer:35S and OsH1.1-Z-R)

Table S1. Cloning and detection primers

Primers	Sequence
OsH1.1-OE-F	5'-tggagaggacagcccaagcttATGCCTGCCATGGCCAAG-3'
OsH1.1-OE-R	5'-gtaccgaattcccggggatccTCAAGCTGCAGGTGCCTTG-3'
OsH1.1-GFP-F	5'-tggagaggacagcccaagcttATGCCTGCCATGGCCAAG-3'
OsH1.1-GFP-R	5'-ctcaccatgaccggtggatccGCTGCAGGTGCCTTGCGG-3'
OsH1.1-GUS-F	5'-gacctgcaggcatgcaagcttATCCTACGTCGAGCCATCCTC-3'
OsH1.1-GUS-R	5'-ttaccctcagatctaccatggCGCAACTTCGCAAGCAGAC-3'
OssHSP24.1-F	5'-TGCTCAAGGTGGTCGTGCCC-3'
OssHSP24.1-R	5'-CCAAAACTTTTATCTCCATAACTT-3'
OsHSP26-F	5'-CCTCGTGGACCCGATGTC-3'
OsHSP26-R	5'-CACCATCACCTTCACCTCCT-3'
OsHSP101-F	5'-GGAGGAGTACCGCAAGTACG-3'
OsHSP101-R	5'-GAGGTCGATCGCTTTGTCAG-3'
OsCBF1-F	5'-GAGACCTTCGCCAACGATG-3'
OsCBF1-R	5'-CACCGGCAACACGTCCTT-3'
OsCBF2-F	5'-TACGGCAACATGGACTTCGA-3'
OsCBF2-R	5'-GCCCATCCCGTCGTAGTAGTAG-3'
OsCBF3-F	5'-AGCGACCTGGCGTTCG-3'
OsCBF3-R	5'-TCGCGTAGTACAGGTCCCA-3'
OsTPP1-F	5'-CCTTCAGCAAATCATGAGCA-3'
OsTPP1-R	5'-AGCCTCCAGCACTTCGTTTA-3'
OsUBQ5-F	5'-ACCACTTCGACCGCCACTACT-3'
OsUBQ5-R	5'-ACGCCTAAGCCTGCTGGTT-3'
OsH1.1-Z-R	5'-gtaccgaattcccggggatccTCAAGCTGCAGGTGCCTTG-3'
35S	5'-CGCACAATCCCACTATCCTTCG-3'
OsH1.1-GUS-F	5'-gacctgcaggcatgcaagcttATCCTACGTCGAGCCATCCTC-3'
GUS-R	5'-CTTCCTCATCCACGACCG-3'
